# Effect of Education on Myopia: Evidence from the United Kingdom ROSLA 1972 Reform

**DOI:** 10.1167/iovs.61.11.7

**Published:** 2020-09-04

**Authors:** Denis Plotnikov, Cathy Williams, Denize Atan, Neil M. Davies, Neema Ghorbani Mojarrad, Jeremy A. Guggenheim, for the UK Biobank Eye and Vision Consortium

**Affiliations:** 1 School of Optometry and Vision Sciences, Cardiff University, Cardiff, United Kingdom; 2 Population Health Sciences, Bristol Medical School, University of Bristol, Bristol, United Kingdom; 3 Medical Research Council Integrative Epidemiology Unit, University of Bristol, Bristol, United Kingdom

**Keywords:** refractive error, myopia, education, regression discontinuity, UK Biobank

## Abstract

**Purpose:**

Cross-sectional and longitudinal studies have consistently reported an association between education and myopia. However, conventional observational studies are at risk of bias due to confounding by factors such as socioeconomic position and parental educational attainment. The current study aimed to estimate the causal effect of education on refractive error using regression discontinuity analysis.

**Methods:**

Regression discontinuity analysis was applied to assess the influence on refractive error of the raising of the school leaving age (ROSLA) from 15 to 16 years introduced in England and Wales in 1972. For comparison, a conventional ordinary least squares (OLS) analysis was performed. The analysis sample comprised 21,548 UK Biobank participants born in a nine-year interval centered on September 1957, the date of birth of those first affected by ROSLA.

**Results:**

In OLS analysis, the ROSLA 1972 reform was associated with a −0.29 D (95% confidence interval [CI]: −0.36 to −0.21, *P* < 0.001) more negative refractive error. In other words, the refractive error of the study sample became more negative by −0.29 D during the transition from a minimum school leaving age of 15 to 16 years of age. Regression discontinuity analysis estimated the *causal effect* of the ROSLA 1972 reform on refractive error as −0.77 D (95% CI: −1.53 to −0.02, *P* = 0.04).

**Conclusions:**

Additional compulsory schooling due to the ROSLA 1972 reform was associated with a more negative refractive error, providing additional support for a causal relationship between education and myopia.

Myopia is a common refractive error causing blurred distance but clear near vision (nearsightedness) because light from distant objects is focused in front of the photoreceptor plane of the retina. The symptoms of myopia can be reduced by optical correction, e.g. spectacles, contact lenses or corneal refractive surgery. However, axial elongation associated with myopia increases the risk of sight-threatening complications such as retinal detachment, choroidal neovascularization and glaucoma.^1^^–^^3^ The prevalence of myopia has increased rapidly over recent decades; however, it varies across geographical location and ancestry.^3^^,^^4^ Currently, 30% or more of the population in Europe and the United States are myopic.^4^^,^^5^ In Singapore, South Korea, and other developed countries of East and South East Asia, the levels are higher, especially in young adults.^4^^,^^6^^–^^8^

Education and outdoor activity are among the environmental risk factors most consistently associated with myopia.^9^^–^^11^ An association between myopia prevalence and educational attainment has been identified in numerous observational studies carried out over more than a century and in many different parts of the world.^4^^,^^7^^,^^12^^–^^14^ Such consistent findings argue in favor of a causal relationship. However the findings from observational studies are potentially biased due to confounding by factors such as socioeconomic position and parental educational attainment. More convincing evidence that education is a *causal* risk factor of myopia is limited to two recent Mendelian randomization studies.^15^^,^^16^

Regression discontinuity is a quasiexperimental design widely used in econometrics research (for an overview of the method, please see Supplementary Note S1). When assignment to different levels of an exposure is based on a continuously measured random variable, the assignment of individuals on either side of some threshold is essentially random.^17^^,^^18^ Raising of the School Leaving Age (ROSLA) was implemented in England and Wales in September 1972.^19^ Children born in September 1957 were the first to be affected by the reform; those who would have left school aged 15 were required to remain at school for up to one additional academic year.^20^ Because ROSLA 1972 generated a marked increase in the duration of education for those affected, it provided a scenario well-suited for regression discontinuity.^21^ In this study, we applied regression discontinuity analysis to estimate the causal association of the ROSLA 1972 reform with refractive error.

## Methods

### Study Sample

We analyzed cross-sectional data from the baseline assessment of the UK Biobank project.^22^ During the period 2006-2010, UK Biobank recruited 502,649 participants aged 37 to 73 years old, who attended one of 22 assessment centers, at which they completed a touch-key questionnaire, had a face-to-face interview with a trained nurse, and underwent physical assessments. The information collected included the participants’ date of birth, ethnicity, country of birth, and educational or professional qualifications. The latter was ascertained with the questionnaire item: “Which of the following qualifications do you have (you can select more than one)?”; with the options, “(1) College or University degree, (2) A levels/AS levels or equivalent, (3) O levels/GCSEs or equivalent, (4) CSEs or equivalent, (5) NVQ or NHD or NHC or equivalent, (6) Other professional qualifications, e.g., nursing, teaching, (7) None of the above”. The age at which continuous full-time education was completed (‘*EduYears*’) was asked only of individuals who reported *not* holding a College or University degree. Therefore, participants with a College or University degree were assigned as having left full time education at the age of 21 years (*EduYears = 21*). Participants who reported completing full time education at age 13 years or less were assigned as having 13 years of educational attainment (*EduYears = 13*).

Toward the latter stages of the recruitment process, an ophthalmic component was added to UK Biobank. Approximately 23% of participants underwent this ophthalmic assessment, which asked about their history of eye disorders and included noncycloplegic autorefraction (Tomey RC5000 autorefractor; performed after removal of habitual spectacles or contact lenses). The refractive error of an individual was calculated as the spherical equivalent (sphere power + 0.5 × cylinder power) averaged between the two eyes.^23^ Sanfilippo et al.^24^ reported that lack of cycloplegia has minimal impact on population estimates of refractive error in individuals older than 20 years of age.

**Figure 1.**
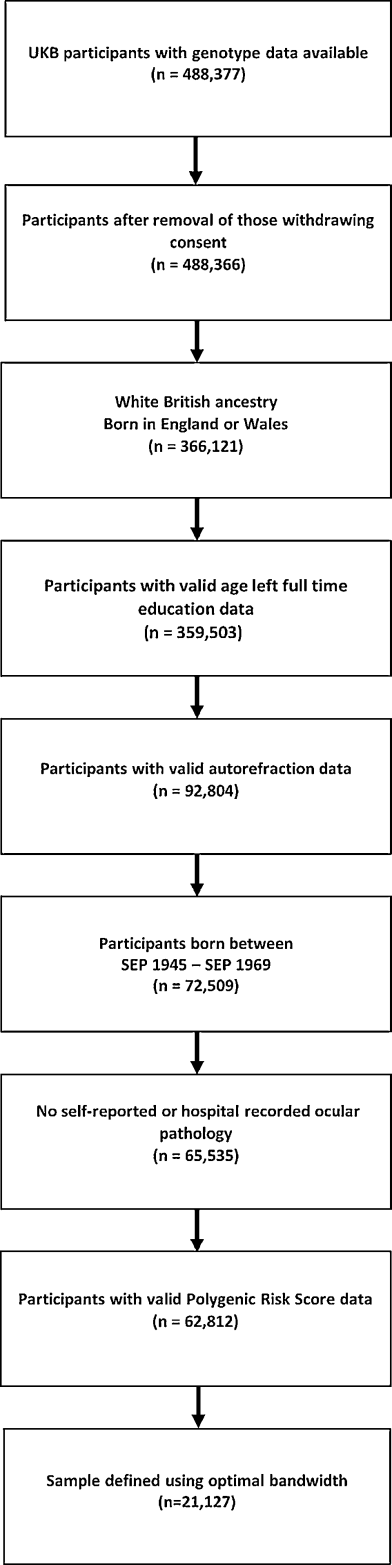
Flow diagram illustrating the selection of UK Biobank participants for the regression discontinuity analysis.

The selection of participants is illustrated in Figure 1. Analysis was restricted to those with data available for refractive error, age completed full-time education, England or Wales as the country of birth, and a known month and year of birth. Participants with a self-reported history of eye trauma resulting in loss of vision, cataract surgery, laser eye surgery or corneal graft surgery were excluded. To avoid population stratification in the genetic component of the study (see below) participants whose genetic ancestry did not cluster with White British individuals were also excluded.^25^ This resulted in a final sample size of 21,127 participants for the main analysis (after selection based on the “optimal bandwidth” method of Calonico et al.,^26^ as described below).

### Genetic Data

Genotyping in UK Biobank sample was performed using either the Axiom or the Affymetrix array.^25^ Genetic data for 488,375 participants were released after rigorous quality control and imputation of SNP not directly genotyped. Summary statistics from a GWAS for age of onset of spectacle wear (AOSW)-inferred spherical equivalent refractive error in 287,448 UK Biobank participants was used to construct a binary polygenic risk scores (PRS) for predicting if an individual had a relatively high or low genetic predisposition to myopia (Supplementary Note S2).^27^ A Bayesian approach that takes into account linkage disequilibrium (LD) between variants was used to determine the weights assigned to variants included in an initial, continuously-scaled PRS for predicting refractive error (implemented in LDpred v1.0.6)^28^ for a set of 1,175,465 HapMap3 variants.^29^ This initial PRS was then standardized and converted it to a binary variable (Supplementary Note S3). The variance in refractive error explained by the binary PRS for high versus low genetic predisposition to myopia was calculated as the increase in the coefficient of determination (*R*²) of a linear regression model with the binary PRS predictor variable included versus the *R*² of a baseline model that comprised the covariates gender, year of birth, and first five ancestry principal components (PC).

### Effect of ROSLA on School Leaving Age

We defined September 1957 as the “cutoff point.” Participants born after this date would have been affected by the ROSLA reform whereas those born earlier would not. Two binary variables were created to examine the proportion of individuals leaving school at the age of ≤15 years before versus after the introduction of the ROSLA reform: First, variable “*leave16*,” which was coded “1” if the participant completed full-time education at age 16 years or above and coded “0” otherwise, and second, variable “*ROSLA*,” coded “1” if a participant was born in September 1957 or later and coded “0” otherwise. A logistic regression was performed to estimate the association of the outcome variable *leave16* with the predictor variable *ROSLA*. The regression was adjusted for gender, month of birth, and first five ancestry PC. Standard errors were clustered by month and year of birth (“*glm.cluster”* command from the *miceadds* R package). The five ancestry PC were included in the model to reduce the influence of population stratification.

### Effect of ROSLA on Refractive Error

A linear regression analysis was used to estimate the effect of ROSLA on refractive error. Specifically, an OLS linear regression was performed in which the outcome variable refractive error was regressed on the binary predictor variable *ROSLA* (as defined above). The regression was adjusted for gender, month of birth, and the first five ancestry PC. Standard errors were clustered by month and year of birth.

The regression discontinuity analysis was carried out as follows. Because not all school pupils were affected by the ROSLA reform, i.e. some people still left school at age 15 or earlier, we performed a “fuzzy” rather than a “sharp” regression discontinuity analysis.^30^ Under this scenario, the results of a regression discontinuity analysis should be interpreted as being restricted to individuals affected by ROSLA (so called “compliers”; see Supplementary Note S1). The birth date of participants (in months) was used as the regression discontinuity “running variable,” in accordance with previous studies investigating the effect of ROSLA on health and socioeconomic outcomes.^31^^–^^33^ The running variable was coded as the number of months before or after the cutoff date that the participant was born; negative values were used for those born before the cut-off date and positive values otherwise. Note that the “bin size” (one month) refers to the sampling units of the running variable.

The “bandwidth” of a regression discontinuity analysis corresponds to the number of months and years before and after the cutoff date that are considered in the analysis. For example, an analysis with a (small) bandwidth of one year would include only the few participants born within one year of the cutoff date, whereas an analysis with a (large) bandwidth of 12 years would include the much larger number of participants born within 12 years of the cutoff date. (In practice, the choice of bandwidth represents a trade-off between the greater precision gained by a larger sample size and the diminishing effect on the discontinuity for participants born more and more distantly from the cutoff date). To determine the optimal bandwidth, we used the selection method of Calonico et al.,^26^ (*“rdbwselect”* function from the R package *rdrobust*). A triangular kernel was used to weight the observations, as proposed by Imbens and Kalyanaraman^34^ (argument kernel = ‘tri’ in *rdrobust* R package).

### Heterogeneity in the Effect of Education on Refractive Error

To test whether the causal effect of ROSLA 1972 on refractive error varied in participants depending on their genetic predisposition to myopia, we stratified the study sample into subsamples with either a relatively high (*n* = 10,548) or low (*n* = 10,579) genetic risk of myopia—based on the binary PRS for refractive error—and estimated the causal effect in each subsample. The regression discontinuity analysis in each subsample was performed using the same method as described in the *Effect of ROSLA on refractive error* section above.

### Inverse Probability Weighting

Due to non-random ascertainment, people who left school at age 15 are underrepresented in the UK Biobank sample. Clark and Royer^31^ reported that 33.0% of participants in the Health Survey for England and the General Household Survey left school at age 15, compared with 17.5% in the UK Biobank. Therefore we used inverse probability weighting (weighting factor = 33/17.5) to correct for the nonrandom sampling in the main analyses.^35^

### Sensitivity Analyses

Tests for discontinuity effects at dates other than the implementation date of ROSLA 1972 were performed, as proposed by Imbens and Lemieux.^36^ Specifically, the association with refractive error was assessed for two “dummy” reforms, namely two years before and two years after September 1972. Note that discontinuity in the refractive error of the study sample at the “dummy” cutoff dates would not be expected, thus providing the opportunity to detect false-positive regression discontinuity effects. McCrary's test^37^ was used to examine the assumption of no manipulation with the running variable (i.e., no discontinuity in the density of the running variable at the cutoff date). To examine the robustness of the regression discontinuity results, the analysis was repeated for a range of different bin sizes (two, three, six, and 12 months) and bandwidths (1–12 years); note that for bin sizes of six and 12 months there were no data points available to fit models with bandwidths of one and two years. The sample size varied with bandwidth; the maximum sample size was *n* = 62,812 for a bandwidth of 12 years.

## Results

### Characteristics of Study Participants

Table 1 summarizes the demographic characteristics of the main analysis sample (*n* = 21,127) and Supplementary Figure S5 illustrates how refractive error varied with year-of-birth in the full sample (*n* = 62,812). The average age of participants was 52.9 years (95% confidence interval [CI]: 52.85 to 52.92), 56.3% were female, and approximately 38.2% had a university or college degree. The proportion of participants who were female, had a university or college degree, and had a relatively high genetic predisposition for myopia did not differ substantially (*P* > 0.05) between those born before versus after the ROSLA reform cutoff date of September 1957. By contrast, there was evidence of a difference in the proportion who wore glasses (94.8% vs. 89.3%, *P* < 0.001), their refractive error (−0.02 vs. −0.15 D, *P* < 0.001), the age they started wearing glasses (40 vs. 35 years old, *P* < 0.001) and their socioeconomic position (Townsend Deprivation Index −2.03 vs. −1.85, *P* < 0.001) between those born before versus after the cutoff date. These observed differences in refractive error-related characteristics and socioeconomic position could potentially have been caused by the longer duration of education for some participants after the introduction of the ROSLA reform. Alternatively, an unmeasured confounding factor—for example, a change in time spent outdoors during childhood over the years in question—could potentially have caused the observed changes in both refractive error and socioeconomic position. It was notable that the proportion of the sample wearing glasses was *lower* after the education reform, which was counterintuitive. We speculate that the lower proportion of the sample wearing glasses after ROSLA compared to before—despite the average refractive error being more negative—may be explained by myopia offsetting the need for reading glasses in some participants.

**Table 1.** Demographic and Genetic Characteristics of the Regression Discontinuity Analysis Sample

Variable	Statistic	All (n = 21,127)	Born Before Cutoff Date (n = 11,556)	Born After Cutoff Date (n = 9571)	*P*
Age	Mean (95% CI)	52.89 (52.85 to 52.92)	54.90 (54.88 to 54.93)	50.45 (50.43 to 50.49)	<2.2E-16
Female	N (%)	11,881 (56.3%)	6539 (56.6%)	5342 (55.9%)	2.70E-01
Wears glasses	N (%)	19,492 (92.3%)	10,956 (94.8%)	8536 (89.3%)	2.30E-51
University or College degree	N (%)	8078 (38.2%)	4462 (38.6%)	3616 (37.8%)	2.20E-01
High genetic predisposition to myopia	N (%)	10,548 (49.9%)	5762 (49.9%)	4786 (50.0%)	8.50E-01
Refractive error (D)	Median (IQR)	−0.08 (−1.60 to 0.71)	−0.02 (−1.54 to 0.85)	−0.15 (−1.70 to 0.54)	3.40E-18
Age started wearing glasses (Years)	Median (IQR)	38.00 (16.00 to 46.00)	40.00 (15.33 to 47.00)	35.00 (16.00 to 45.00)	1.00E-12
Townsend Deprivation Index	Median (IQR)	−1.95 (−3.51 to 0.51)	−2.03 (−3.55 to 0.44)	−1.85 (−3.44 to 0.64)	9.40E-04

Participants were stratified based on being born before or after the cutoff date, i.e. not affected vs. affected by the ROSLA reform.

We investigated whether individuals in the RD sample born before the cutoff differed in their demographic characteristics depending on their having a relatively high versus low genetic susceptibility to myopia (Table 2). Individuals with high genetic risk for myopia development had a more negative refractive error (median −0.47 D vs. 0.31 D), an earlier age of onset of spectacle wear (29 vs. 40 years), and were more highly educated (41.8 % vs. 35.4% with a university or college degree).

**Table 2.** Demographic and Genetic Characteristics of the Regression Discontinuity Analysis Sample for Individuals Born Before the Cutoff (*n* = 11556)

Variable	Statistic	All (*n* = 11,556)	High Genetic Risk of Myopia (*N* = 5762)	Low Genetic Risk of Myopia (*N* = 5794)	*P*
Age	Median (IQR)	54.92 (53.75 to 56.00)	54.92 (53.83 to 56.00)	54.92 (53.75 to 56.00)	3.50E-01
Female	N (%)	6539 (56.6%)	3285 (57.1%)	3254 (56.2%)	3.40E-01
Wears Glasses	N (%)	10,956 (94.8%)	5476 (95.1%)	5480 (94.6%)	3.10E-01
University or College degree	N (%)	4,462 (38.6%)	2,411 (41.8%)	2,051 (35.4%)	1.30E-12
Refractive error (D)	Median (IQR)	−0.02 (−1.54 to 0.85)	−0.47 (−2.53 to 0.50)	0.31 (−0.66 to 1.16)	<1.0E-99
Age started wearing glasses (Years)	Median (IQR)	40.00 (15.33 to 47.00)	29.00 (13.00 to 45.00)	41.00 (19.00 to 48.00)	6.20E-55
Townsend Deprivation Index	Median (IQR)	−2.03 (−3.55 to 0.44)	−1.99 (-3.56 to 0.41)	−2.06 (−3.54 to 0.47)	8.80E-01

Participants were stratified based on the binary PRS for myopia, i.e., with high or with low genetic risk for myopia.

### Effect of ROSLA on School Leaving Age

**Figure 2.**
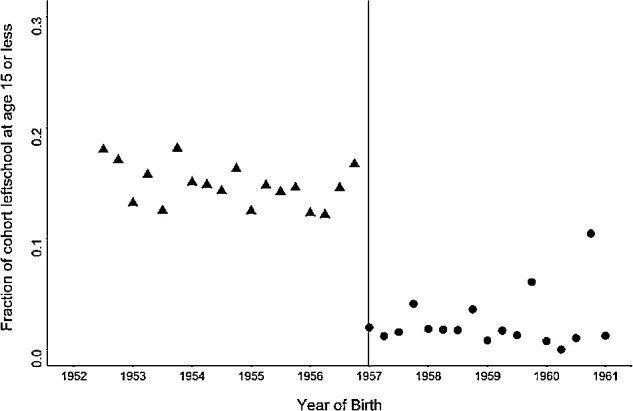
The association of the ROSLA 1972 education reform with the proportion of participants reporting completion of full-time education at age 15 years or younger. *Points* represent the mean for each forthcoming year of birth (running from September to September). The *vertical dotted line* represents the September 1957 cutoff date denoting the month and year of birth for those first affected by ROSLA 1972. Individual outcomes are grouped in three-month bins.

**Figure 3.**
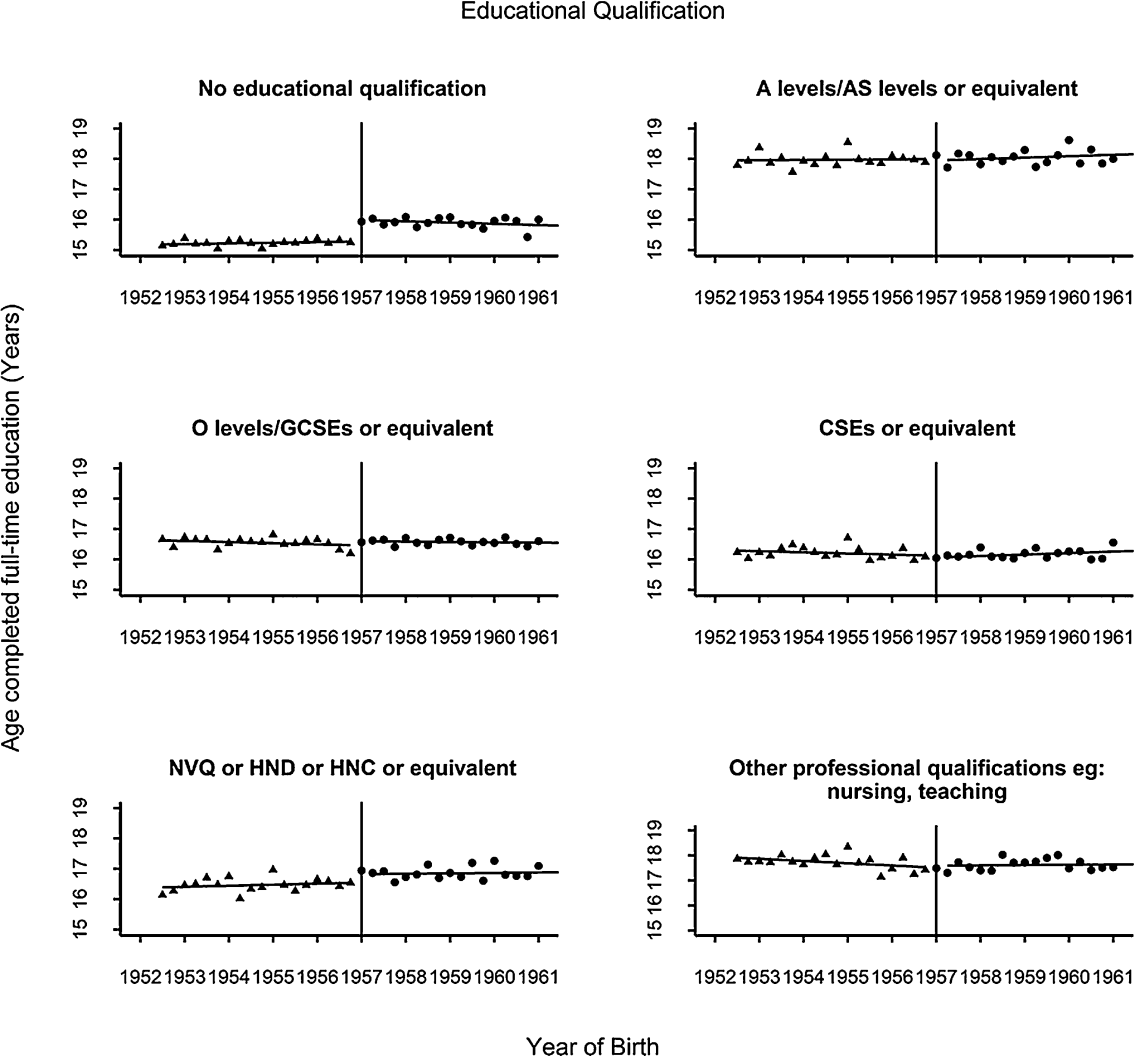
Discontinuity in age completing full time education for participants stratified by highest educational qualification. *Triangles* represent participants born before the cutoff date; *circles* represent participants born after the cutoff date. Individual outcomes are grouped in three-month bins. *Solid black lines* represent the lines of best fit from the linear regression of educational attainment on the running variable.

As shown in Figure 2, the ROSLA reform coincided with an abrupt fall in the proportion of individuals reporting that they completed their education at age ≤15 years, which stabilized thereafter. When analyzed quantitatively, there was a 12.5% (95% CI: 11.8–13.2) reduction in the percentage of those leaving school at age 15 or younger (from 14.9% before the cutoff date to 2.4% after). Stratifying the sample by highest educational qualification indicated a greater impact of ROSLA 1972 in those reaching adulthood with no qualifications compared to participants who attained educational qualifications (Fig. 3) consistent with previously reported findings.^20^^,^^21^

### Effect of ROSLA on Refractive Error

In an OLS regression analysis, the ROSLA 1972 reform was associated with a −0.29 D (95% CI: −0.36 to −0.21, *P* < 0.001) more negative refractive error, after statistically adjusting for gender, month of birth, and the first five genetic ancestry PC. That is, on average, individuals born after the cutoff date of September 1957 had a −0.29 D more negative refractive error in adulthood than those born before the reform. This result supported the theory that additional time spent in education increases the risk of myopia. However, as emphasized above, an OLS estimate such as this is at high risk of bias from confounding factors. Therefore, to provide an estimate of the *causal* effect of the ROSLA reform on refractive error, we performed a regression discontinuity analysis. Using the “optimal bandwidth” of 53.6 months (4.5 years) either side of the cutoff date, the estimated causal effect was −0.77 D (95% CI: −1.53 to −0.02, *P* = 0.04). Thus the regression discontinuity supported the hypothesis that additional time spent in education is causally associated with a more negative refractive error, on average.

### Heterogeneity Relating to Genetic Predisposition to Myopia

The binary PRS for predicting high versus low genetic predisposition for myopia explained 4.1% (*P* < 0.001) of the variance in refractive error. We used the binary PRS to stratify participants in the analysis sample into groups with relatively high or low genetic predisposition to myopia development. In the group with a high genetic predisposition to myopia, the regression discontinuity analysis provided little support for a non-zero effect: Causal effect estimate = −0.50 D (95% CI: −1.34 to 0.23, *P* = 0.60), whereas in the group with a low genetic predisposition to myopia the causal effect estimate was relatively large: Causal effect estimate = −1.47 D (95% CI: −2.81 to −0.12, *P* = 0.03). The corresponding OLS regression estimates in the two groups were -0.30 D (95% CI: −0.41 to −0.19, *P* < 0.001) and −0.25 D (95% CI: −0.34 to −0.16, *P* < 0.001), respectively. However, tests for differences in the effect size across the two strata were not supportive of a meaningful level of heterogeneity (independent samples *t*-test, *P* = 0.09 and *P* = 0.55 for the regression discontinuity and OLS analysis, respectively).

### Sensitivity Analyses

Arbitrary cut-off dates of two years before or two years after ROSLA were not associated with discontinuities in either educational attainment or refractive error and yielded causal effect estimates close to zero (*P* = 0.57 and *P* = 0.62, respectively). There was little evidence of “running variable manipulation” around the cutoff date (McCrary density test for bin size one month, *P* = 0.21), suggesting that parents did not intentionally favor August or September as the month of birth. Regarding the robustness of the results to the exact choice of bandwidth and bin size, the regression discontinuity estimates showed consistency in the magnitude and the direction of effect across a wide range of bin sizes and bandwidths (Fig. 5).

## Discussion

The ROSLA 1972 educational reform was used as a natural experiment in a regression discontinuity framework to investigate the causal relationship between education and refractive error. The point estimate of the causal effect of ROSLA 1972 suggested a more negative refractive error in UK Biobank participants affected by the reform. Specifically, the regression discontinuity causal relationship estimate was −0.77 D in the direction of myopia (95% CI: −1.53 to −0.04, *P* = 0.04).

Using a binary PRS to stratify the cohort by the level of genetic risk of myopia, there was suggestive evidence of heterogeneity in the association. The results suggested that those at high genetic risk of myopia may have been affected by ROSLA to a lesser extent than those at low genetic risk of myopia. If such heterogeneity is genuine, it may indicate that genetically-predisposed individuals are likely to develop myopia irrespective of their exposure to education. Providing indirect support for this result, Pozarickij et al.^38^ recently demonstrated that 88% of known GWAS variants associated with refractive error exhibited evidence of interaction effects, including gene × education interaction effects.

A strength of the regression discontinuity design is that participants born just before or just after the cutoff date differ only in their treatment assignment, and not—at least in theory—in exposure to confounders.^39^ A further strength of the study was that we were able to restrict the sample to participants whose genetic ancesty clustered with white British individuals, in order to limit the influence of population stratification. Consistent with expectations, there was no appeciable difference in genetic predisposition to myopia in participants born before versus after the cutoff date (Table 1).

**Figure 4.**
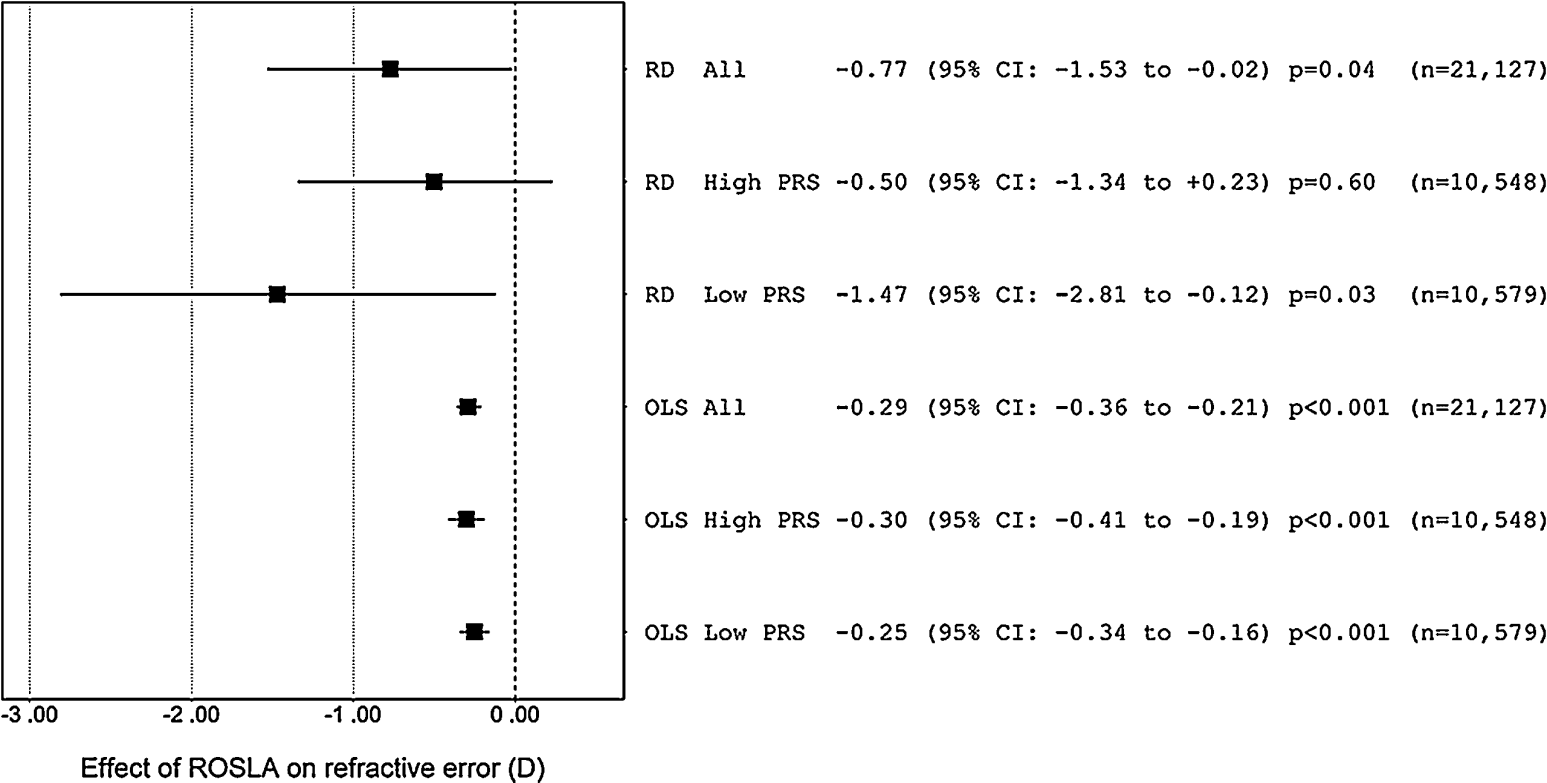
Effect estimate of the ROSLA 1972 education reform on refractive error obtained using regression discontinuity analysis and OLS regression. Results are presented for the full sample or separately for those with a high genetic predisposition (High PRS) or a low genetic predisposition (Low PRS) of myopia based on a binary polygenic risk score.

**Figure 5.**
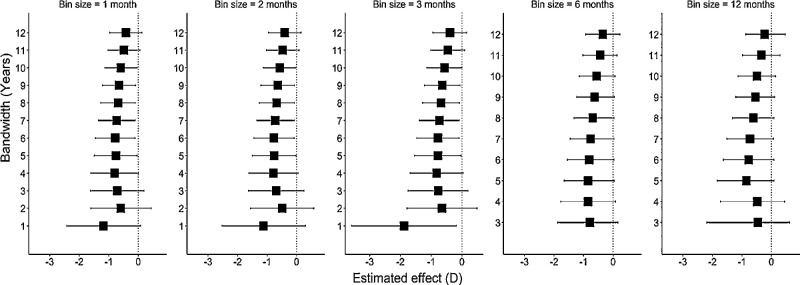
Causal effect estimates of the ROSLA 1972 educational reform obtained using regression discontinuity analysis for different bin sizes at a range of different bandwidths.

Key limitations of the study were the possibility of selection bias and the modest sample size. The difference of the UK Biobank participants from the general population with regard to educational attainment^40^ could potentially generate a spurious association even in the absence of a causal relationship between the exposure and the outcome.^41^ We applied inverse probably weighting to account for the excess of highly educated individuals in UK Biobank. This had a large impact on the results: The causal effect estimated without inverse probably weighting was −0.55 D (95% CI: −1.80 to 0.70, *P* = 0.39); this was lower and had a much wider confidence interval than the estimate of −0.77 D (95% CI: −1.53 to −0.04, *P* = 0.04) after inverse probably weighting. The sample size of our analyses was constrained to restrict attention to participants born close to the cutoff date. This led to low precision in the estimated effect of education on refractive error (Figs. 4 and 5). Future studies in larger samples or assessing the effects of other schooling reforms would be helpful. Another possible limitation is that educational attainment^42^ and refractive error^43^ vary with month of birth (Supplementary Note S4), and therefore could bias the casual effect estimate. However, sensitivity analyses using a 12-month bin size, which would have smoothed out month-of-birth influences such that they would not contribute to the discontinuity at the ROSLA cutoff date, produced a comparable causal effect estimate. The similarity of the causal effect estimates across a range of bin sizes also argues against a major source of bias from month of birth-related associations (Fig. 5).

Our results support the findings from Mendelian randomization studies that remaining in education *causally* increases the risk of myopia. However, the mechanism underlying this relationship remains unclear. In the current study, we estimated the effect of the *educational reform* per se and the causal effect was restricted to the effect in individuals affected by the reform. Therefore our causal effect estimate encompasses the effect of changes in educational attainment for individuals born just before versus after the cutoff date, but not the effect of an additional year in education. This is the first study to estimate the effect of ROSLA 1972 on refractive error using a quasiexperimental approach.

In summary, a regression discontinuity analysis provided evidence of an association between education and refractive error consistent in direction and magnitude with that obtained by OLS regression, suggesting that the shift in refractive error towards myopia associated with higher educational attainment results from a causal relationship. Specifically, the ROSLA 1972 education reform was associated with a more negative refractive error within the range of −1.53 to −0.02 D. There was suggestive evidence that the magnitude of the association was higher in individuals with a relatively low genetic predisposition to myopia compared to those with a high genetic predisposition. This work supports the findings from Mendelian randomization studies implicating education as a causal risk factor for myopia.

## Supplementary Material

Supplement 1
