# Supplementary material for: Effect of Education on Myopia: Evidence from the United Kingdom ROSLA 1972 Reform
Source: Invest Ophthalmol Vis Sci. 2020 Sep 4;61(11):7. doi: 10.1167/iovs.61.11.7 (PMC7476669; doi:10.1167/iovs.61.11.7)
Supplement: Supplement 1 [file iovs-61-11-7_s001.pdf]

Supplementary Material

Effect of education on myopia: Evidence from the United Kingdom ROSLA 1972 reform

Plotnikov et al.

Contents	Title	Page
Supplementary Note 1	Key concepts of Regression discontinuity analysis.	2
Supplementary Figure S1	Key concepts of Regression discontinuity analysis.	4
Supplementary Note 2	Validity of a polygenic risk score (PRS) for refractive error derived from a GWAS for age-of-onset of spectacle wear (AOSW)-inferred refractive error.	5
Supplementary Figure S2	Miami plot of GWAS for refractive error in UK Biobank measured by autorefraction and GWAS for AOSW-inferred refractive error.	6
Supplementary Note 3	PRS for high vs. low genetic predisposition for myopia.	7
Supplementary Note 4	Relationship between month-of-birth and education, and between month-of-birth and refractive error.	8
Supplementary Figure S3	The association of month of birth with refractive error in the RD sample (n = 21,217).	9
Supplementary Figure S4	The association of month of birth with years spent in full-time education in the RD sample (n = 21,217).	9
Supplementary Figure S5	The association of year of birth with refractive error in the full sample (n = 62,812).	10

Supplementary Note 1. Key concepts of Regression discontinuity analysis

Regression discontinuity (RD) analysis is a quasi-experimental design that allows the researcher to estimate the causal effect of an intervention (treatment) so long as the treatment is assigned based on some continuous variable (called the "assignment variable" or "running variable"). In this scenario, treatment assignment is solely dependent on whether an individual's value for the running variable is above or below some threshold level, called the "cut-off" value. Participants just above or below the cut-off are assumed to differ only by the value of the running variable. Within a small window above and below the cut-off value (referred to as the "bandwidth") participants are assumed to be comparable with respect to confounding variables, as further discussed below.

Supplementary Figure 1 illustrates the difference between the RD design and a simple comparison of the mean value of the running variable in the groups above and below the cut-off. In this example, a treatment was assigned for those with the value of a biological parameter (axis X, running variable) with X > 150 units. Individuals on the left side of the cut-off did not receive the treatment and were therefore assigned to the control group. Those on the right side of the cut-off did receive the treatment. As assignment to the treatment was based only on the value of the running variable, then so long as individuals had no control over this variable, i.e. they could not manipulate the running variable, then assignment to the treatment or control groups would have been at random for those extremely close to the cut-off.[1,2] Hence, individuals with values of the running variable extremely close to the cut-off would be expected to be similar in the distribution of confounders (both measured and unmeasured).[3] This similarity of individuals with values of the running variable close to the cut-off permits a valid statistical comparison in which any difference in the outcome (axis Y, some health effect) is due to the treatment, i.e. a causal effect of the treatment, rather than to the effects of confounders. Individuals far from the cut-off are not similar as regards the distribution of confounders, therefore the direct comparison of the mean effect in the treatment vs. control groups may lead to an invalid causal effect estimate. One of the key aspects of RD is the trade-off inherent in the choice of the optimal bandwidth. A bandwidth that is narrow will provide a more even distribution of confounders in participants either side of the cut-off, while a wider bandwidth will provide a larger sample size and thus yield greater statistical power and a more precise causal effect estimate.

The two main types of RD design described in the literature are the "sharp" and "fuzzy" designs.[4] In the sharp design, all individuals with values of the running variable higher than the cut-off value are assigned to receive the treatment, and those with running variable values less than the

cut-off are assigned to the control group. Hence, assignment is a deterministic function of the running variable.[5] In the fuzzy RD design, assignment is a probabilistic function of the running variable such that less than 100% of individuals with values of the running variable above the cut-off are assigned to the treatment.[6] The fuzzy RD design works under the assumption that some of the individuals receiving the treatment would not have done so in the absence of the assignment. This subgroup of treated individuals are called "compliers".[7]

With regards to the ROSLA reform, compliers are those individuals who were legally obliged to stay in school by the reform and did so. Hence, excluding people with qualifications from an analysis sample investigating the effects of ROSLA risks excluding those individuals who stayed in school due to the reform, i.e. the compliers. As these are precisely the individuals of most interest, this will introduce bias into the causal effect estimate. Likewise, stratification of a ROSLA RD analysis sample based on highest educational qualification may introduce selection bias (a form of collider bias) which may distort the association between the intervention (ROSLA) and an outcome such as refractive error.[8]

Supplementary Figure S1. Key concepts of Regression discontinuity analysis. Black solid lines represent realized outcomes before and after the treatment; the black dotted line represents the (counterfactual) outcome that would have occurred without treatment. The thin vertical line represents the cut-off, which determines which individuals receive the treatment.

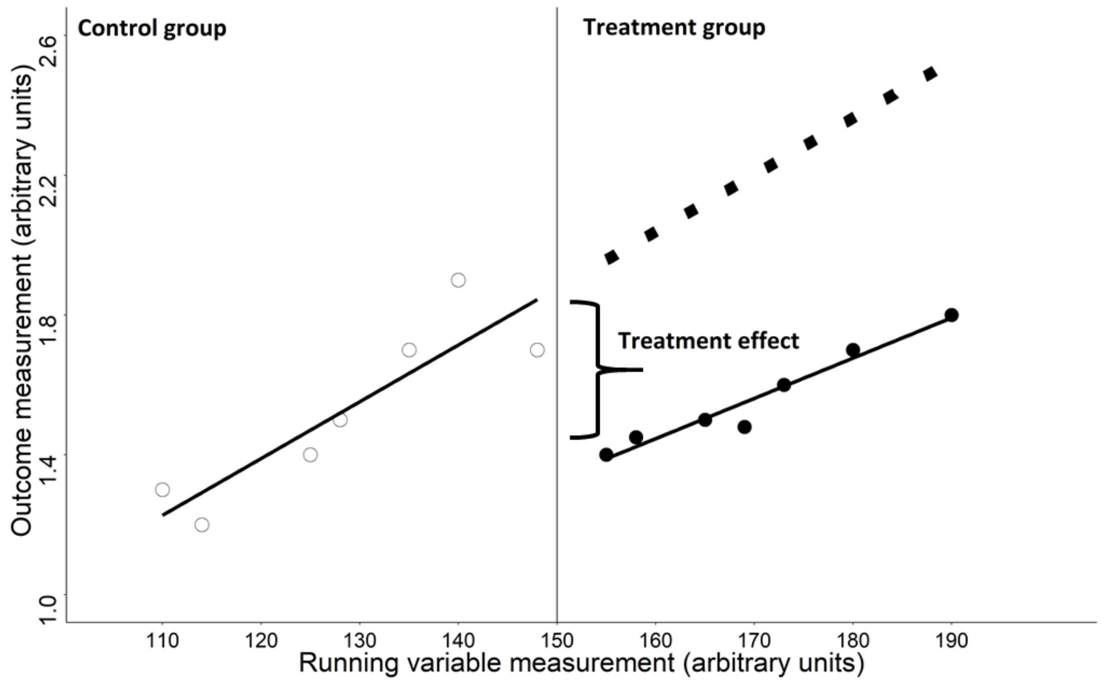

Supplementary Note 2. Validity of a polygenic risk score (PRS) for refractive error derived from a GWAS for age-of-onset of spectacle wear (AOSW)-inferred refractive error.

The SNP weights for a PRS are derived from a GWAS. Since the number of SNPs tested in a GWAS is typically far in excess of the sample size, this can lead to over-fitting. To avoid over-fitting, we obtained SNP weights for our PRS using an independent sample of participants – namely, UK Biobank participants who did *not* undergo autorefraction but who did report their AOSW.

We created the surrogate phenotype 'AOSW-inferred refractive error' as described[9]. Certain values of AOSW are more informative than others in inferring refractive error, for example an AOSW > 40 years-old will generally reflect correction of presbyopia, an AOSW between 10 and 20 years old will typically reflect correction of myopia, while an AOSW < 5 years-old will generally reflect correction of hypermetropia. The statistical model for assigning AOSW-inferred refractive error took these non-linear relationships and probabilities into account. For any individual person, such an inference would be error prone. However, on average, when tested in an out-of-the-bag sample, the AOSW-inferred refractive error was moderately correlated with the true refractive error ($r = 0.55$)[9]. Results of a GWAS for autorefraction-measured refractive error in 95,619 UK Biobank participants and a GWAS for AOSW-inferred refraction error in 287,448 UK Biobank participants are shown in Supplementary Figure S2. This figure illustrates the similarity in the magnitude and direction of effect of the most highly significant genetic variants associated with each trait. This similarity was quantified by calculating the genetic correlation for the two traits[9] (using LD score regression analysis for summary statistics from the above two GWAS). The genetic correlation was $r_g = 0.92$, confirming that genetic risk for AOSW-inferred refractive error is shared with genetic risk for autorefraction-measured refractive error. In summary, we concluded that the PRS for refractive error derived from a GWAS for AOSW-inferred refractive error was a valid PRS for refractive error.

Supplementary Figure S2. Miami plot of GWAS for refractive error in UK Biobank measured by autorefraction (top, n = 95,619) and GWAS for AOSW-inferred refractive error (bottom, n = 287,448). See Ghorbani Mojarrad et al.[9] for details.

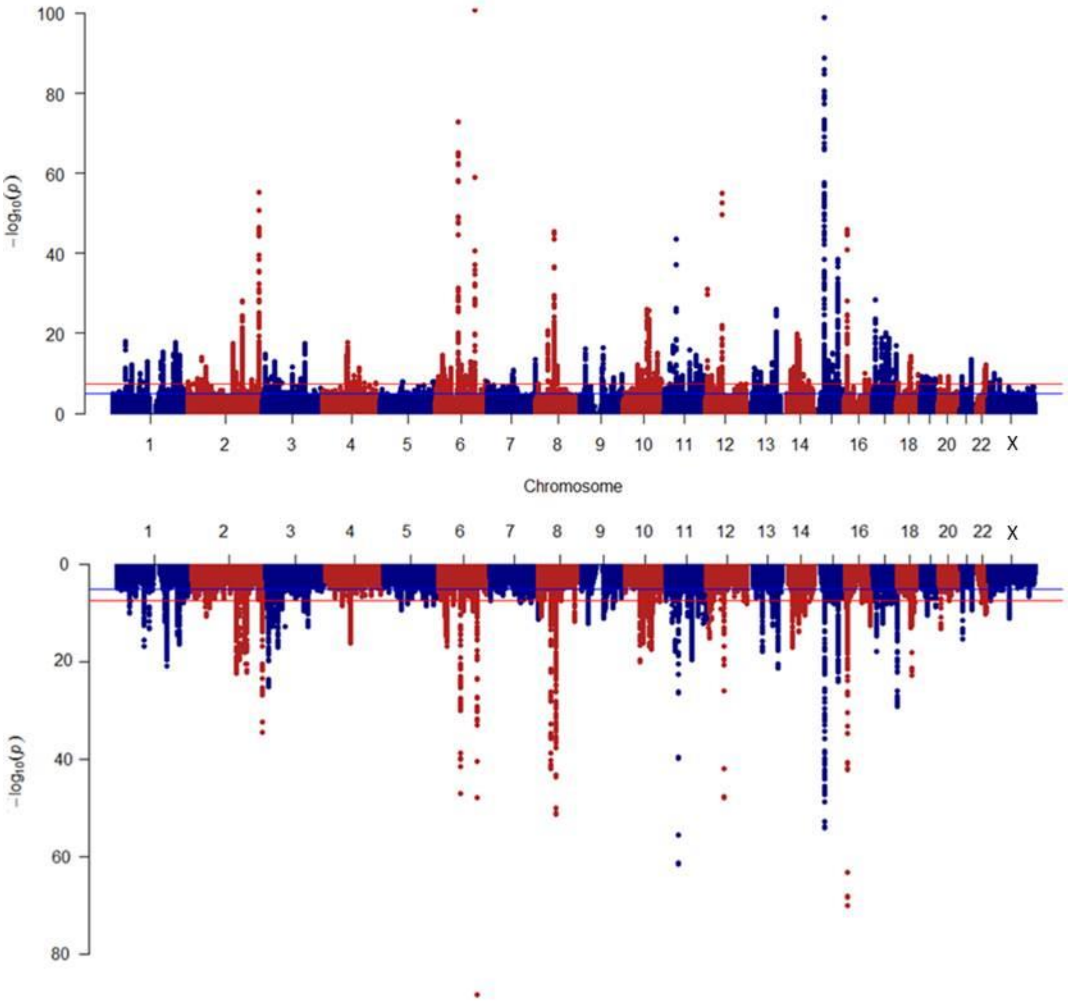

Supplementary Note 3. PRS for high vs. low genetic predisposition for myopia

A PRS for AOSW-inferred refractive error was constructed using a set of ~ 1.1 M genetic variants, as described above and in Ghorbani Mojarrad et al.[9]. This PRS was standardised (to have mean = 0 and variance = 1) and then converted to a binary variable which was equal to 1 if the standardised PRS for refractive error was less than zero; and 0 otherwise. Thus, a value of 1 for this binary variable indicated a relatively high genetic risk of myopia (i.e. a negative refractive error) while a value of 0 indicated a relatively low risk of myopia. The binary PRS for high vs. low genetic predisposition for myopia explained 4.1% ($p < 0.001$) of the variance in autorefraction-measured refractive error in an independent sample (see main text). Our indirect approach of using a GWAS for *AOSW-inferred refractive error* to derive weights for the initial PRS, rather than a GWAS for *AOSW-inferred myopia*, was selected to improve the prediction accuracy of the final binary PRS (since a GWAS for a continuous trait provides greater statistical power than a GWAS for a binary trait).

Supplementary Note 4. Relationship between month-of-birth and education, and between month-of-birth and refractive error.

Refractive error and myopia vary by month (or season) of birth.[10,11] If education is a causal risk factor for myopia then this relationship will be due at least in part to the well-known relationship between month-of-birth and educational attainment, in which the older children in a year-group tend to outperform their younger peers.[12,13]

As illustrated in Supplementary Figures S4 and S5, the pattern of association of refractive error and years spent in full-time education by month of birth in the RD sample was not the same. This suggests that factors relating to month-of-birth in addition to education may exert effects on ocular refraction.

Supplementary Figure S3. The association of month of birth with refractive error in the RD sample (n = 21,217). Months 1-12 correspond to Jan-Dec. Error bars represent 95% confidence intervals.

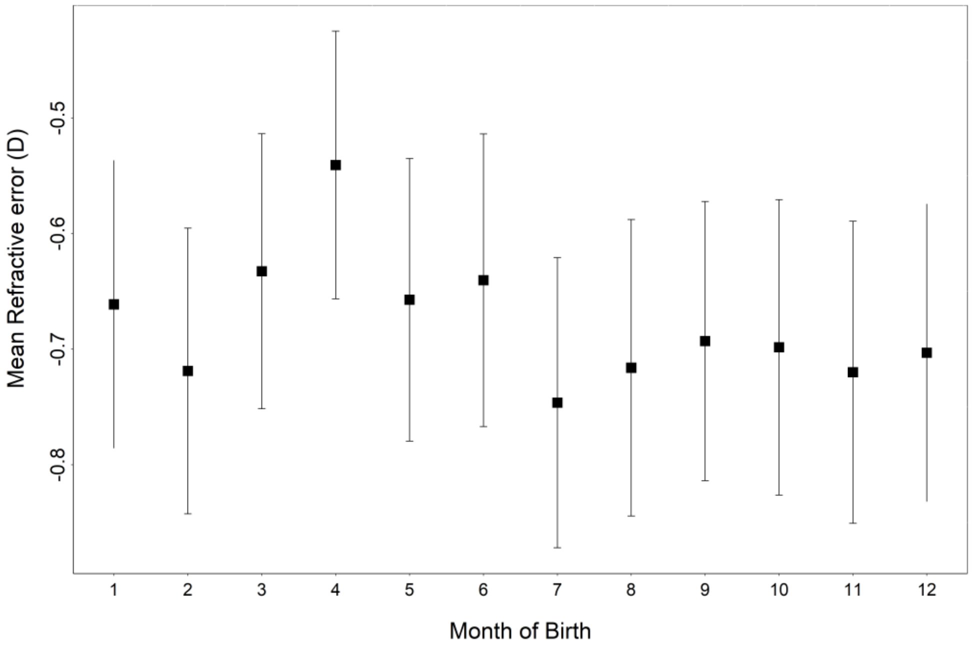

Supplementary Figure S4. The association of month of birth with years spent in full-time education in the RD sample (n = 21,217). Months 1-12 correspond to Jan-Dec. Error bars represent 95% confidence intervals.

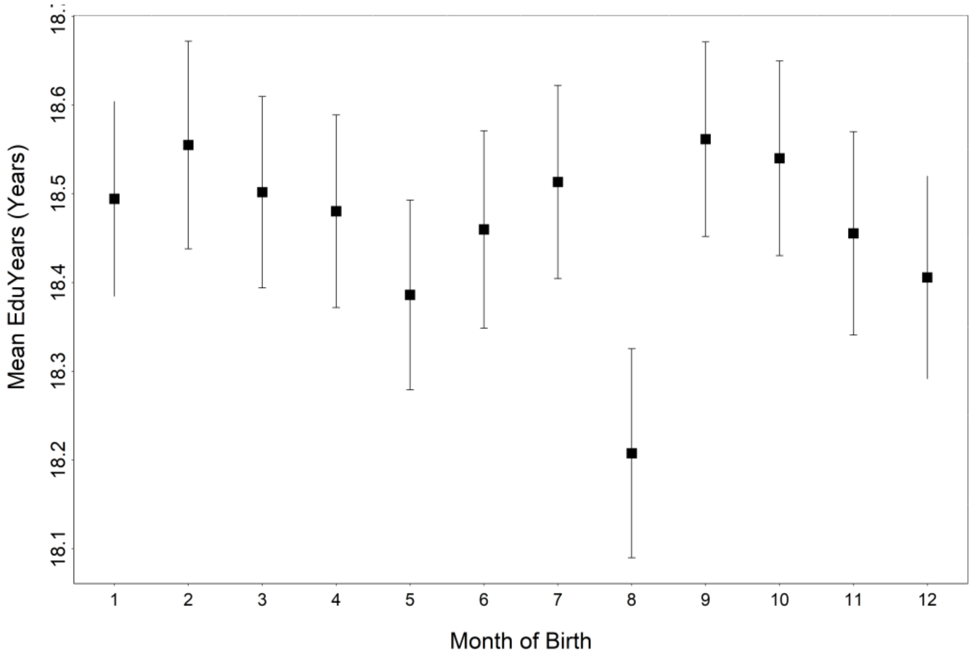

Supplementary Figure S5. The association of year of birth with refractive error in the full sample (n = 62,812). Error bars represent 95% confidence intervals.

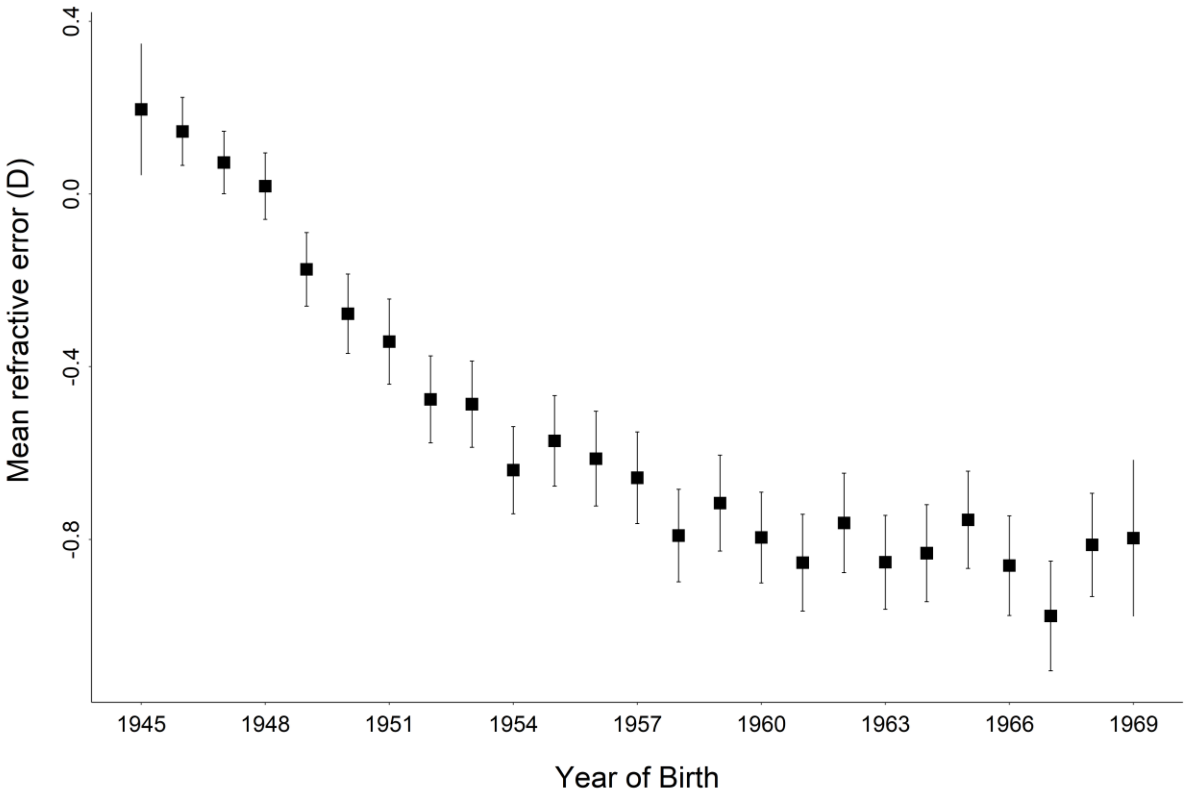

Supplementary References

1. Lee DS, Lemieuxa T. Regression Discontinuity Designs in Economics. *J. Econ. Lit.* 2010;48**:**281-355.

2. McCrary J. Manipulation of the running variable in the regression discontinuity design: A density test. *J Econometrics* 2008;142**:**698-714.

3. Oldenburg CE, Moscoe E, Bärnighausen T. Regression Discontinuity for Causal Effect Estimation in Epidemiology. *Current Epidemiology Reports* 2016;3**:**233-241.

4. Jacob R, Zhu P, Somers MA, Bloom H. A Practical Guide to Regression Discontinuity. New York: MDRC Working Paper MDRC; 2012.

5. Imbens GW, Lemieux T. Regression discontinuity designs: A guide to practice. *J Econometrics* 2008;142**:**615-635.

6. Hahn J, Todd P, Van der Klaauw W. Identification and Estimation of Treatment Effects with a Regression-Discontinuity Design. *Econometrica* 2001;69**:**201-209.

7. Imbens GW, Angrist JD. Identification and Estimation of Local Average Treatment Effects. *Econometrica* 1994;62**:**467-475.

8. Greenland S. Quantifying biases in causal models: Classical confounding vs collider-stratification bias. *Epidemiol.* 2003;14**:**300-306.

9. Ghorbani Mojarrad N, Plotnikov D, Williams C, Guggenheim JA, U.K. Biobank Eye & Vision Consortium. Association Between Polygenic Risk Score and Risk of Myopia. *JAMA Ophthalmol* 2020;138**:**7-13.

10. Mandel Y, Grotto I, El-Yaniv R, et al. Season of birth, natural light, and myopia. *Ophthalmology* 2008;115**:**686-692.

11. McMahon G, Zayats T, Chen Y-P, et al. Season of birth, daylight hours at birth and high myopia. *Ophthalmology* 2009;116**:**468–473.

12. Russell RJH, Startup MJ. Month of birth and academic achievement. *Pers. Individ. Dif.* 1986;7**:**839-846.

13. Kihlbom M, Johansson SE. Month of birth, socioeconomic background and development in Swedish men. *J. Biosoc. Sci.* 2004;36**:**561-571.
